# Regulations, biosecurity measures, and impact of COVID-19: A comprehensive mixed method study in traditional wet and live animal markets in Bangladesh

**DOI:** 10.1016/j.onehlt.2025.101014

**Published:** 2025-03-17

**Authors:** Sukanta Chowdhury, Tushar Kumar Das, Nurun Nahar Chisty, Sajal Kanti Biswas, Mohammed Ziaur Rahman, Jiaxin Ling, Mahmoud M. Naguib, Johanna F. Lindahl

**Affiliations:** a International Centre for Diarrhoeal Disease Research, Bangladesh (icddr,b), Dhaka, Bangladesh; b Zoonosis Science Centre, Department of Medical Biochemistry and Microbiology, Uppsala University, Uppsala, Sweden; c Department of Infection Biology and Microbiomes, Institute of Infection, Veterinary and Ecological Sciences, University of Liverpool, Liverpool, United Kingdom; d Department of Animal Health and Antibiotic Strategies, Swedish Veterinary Agency, Uppsala, Sweden

**Keywords:** Wet and live animal markets, Regulations, Biosecurity, Disease transmission, Food security

## Abstract

**Background:**

Traditional wet and live animal markets (TWLAMs) offer fresh vegetables, meat, fish, and live animals to consumers at affordable prices. The daily operation of TWLAMs is crucial for supplying safe food by controlling and preventing contamination from food-borne pathogens.

**Objectives:**

A cross-sectional study was conducted in 10 TWLAMs to collect and assess data on market regulations, biosecurity measures, and the impact of COVID-19 on food supply and livelihoods.

**Methods:**

We interviewed 40 key informants and performed on-site observations. Additionally, we organized a workshop with different stakeholders including experts in human health, animal health, food safety, zoonotic diseases, agriculture, consumer rights, and market regulations.

**Results:**

Among the 10 surveyed TWLAMs, five (50 %) were governed by the City Corporation, six (60 %) had written operational guidelines, while 40 % were unaware of any government regulations. Most markets (80 %, *n* = 8) lacked direct water supply lines for their shops, and 50 % had no functional drainage systems. A majority (55 %, *n* = 22) of the key informants reported not seeing any food inspectors at the market within the last three months. Only 60 % (*n* = 24) believed that live animals could transmit diseases to humans within wet markets. While shop-level cleaning was regularly conducted, cleaning of the entire market was infrequent. COVID-19 had both negative and positive impacts on TWLAM. The most common negative effects were job losses (65 %) and increased living expenses (67 %), while COVID-19 led to improvements in market hygiene (100 %), personal hygiene (100 %), and adherence to social distancing (100 %). No permanent closures or bans on animal trading were reported in TWLAM during the COVID-19 pandemic. All workshop participants (*n* = 55) indicated that shifting from live animal trading to processed animal products would be challenging due to cultural norms and practices.

**Conclusions:**

Despite many challenges and shortcomings, a unique operational guideline could help ensure the supply of safe food to consumers. Financial incentives, certification, training, and regular monitoring can improve practices associated with food safety.

## 1 Introduction

Traditional wet and live animal markets (TWLAMs) meet the demand for everyday essentials foods such as fresh vegetables, fish, live animals, animal products (meat and egg) and groceries in many countries [[1], [2], [3]]. In Bangladesh, TWLAMs are known as “Kacha Bazar”, where various food products and services are exchanged among merchandisers, shop owners, and consumers [4]. TWLAMs are distributed across the country with a high concentration in urban areas. The majority of urban TWLAMs have permanent infrastructure, whereas most of the rural TWLAMs assemble in open places once or twice per week. Agricultural produce, live animals, and animal products are often sold together in same place [5]. The local governments are the supreme regulatory authority for most of the TWLAMs in both urban and rural areas [5]. In most instances, the daily operations of TWLAMs are controlled and monitored by a management committee which includes representatives from shop owners, local government representatives, civil society members, and market leaseholders [5]. Bangladesh government has a law “Hats and Bazars (Establishment and Acquisition) Act, 2023” to regularize wet, village, animal and food markets [6]. Under the jurisdiction, city corporation, district, sub-district, Pourasava and union council offices form local market management committee for varieties of food, animal markets and other markets to manage toll, value added tax, fees, maintenance and improvement costs of markets [7].

TWLAM is considered as a potential hotspot for zoonotic and food-borne pathogens [1,8]. The trading of live animals and unsafe slaughtering practices with poor hygiene have contributed to the spillover of several pathogens, including highly pathogenic avian influenza virus (HPAIV) H5N1, severe acute respiratory syndrome coronavirus-1 (SARS-CoV-1), and SARS-CoV-2, from animals to humans [[8], [9], [10]]. In addition, improper handling and storage of food leads to food contamination with food-borne pathogens such as *Campylobacter* spp., *Salmonella enterica*, *Escherichia coli*, and *Listeria monocytogenes* [9]. TWLAM is one of the densely populated areas where highly transmissible respiratory pathogens such as human influenza virus and SARS-CoV-2 can quickly spread from person to person. Adherence to the appropriate food safety regulations, guidelines, or standard operating procedures (SOPs), along with improved biosecurity practices, proper respiratory etiquette, personal hygiene, and regular sanitary inspections, plays a crucial role in controlling transmission, spread and contamination by zoonotic and food-borne pathogen. Hence, this study was aimed to collect and assess data on regulations, biosecurity measures, and the impact of the COVID-19 pandemic on food supply and livelihoods in selected TWLAMs.

## 2 Methodology

### 2.1 Study sites and population

**Fig. 1:**
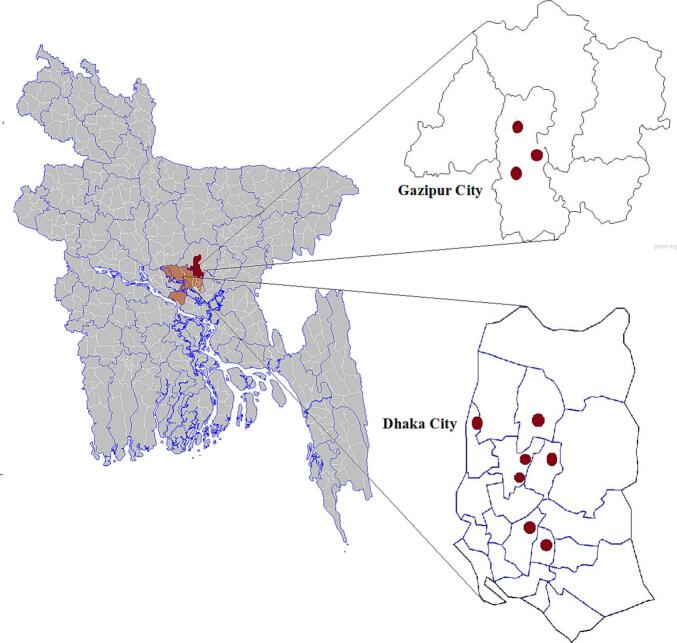

We conducted a cross-sectional study, incorporating both quantitative and qualitative approaches, in permanent TWLAMs in Dhaka and Gazipur city from October to December 2023. Data was collected from a total of 10 TWLAMs: three in Dhaka South City Corporation, four in Dhaka North City Corporation, and three in Gazipur City Corporation (Fig. 1). Dhaka and Gazipur were chosen for this study due to the high concentration of TWLAMs in these areas. Study participants included market committee representatives, poultry vendors, consumers, and City Corporation representatives. Respondents aged 18 years and older, regardless of gender, were enrolled for interviews after informed written consent.

### 2.2 Key informant interviews and data collection

A study team, including a veterinarian and a sociologist, conducted quantitative and qualitative interviews to gather information on market regulations or guidelines, market management, biosecurity practices, personal hygiene, and the impact of COVID-19 on food supply and livelihoods. A total of 40 key informants were purposively selected from 10 markets. In each market, we interviewed a market committee member, a vendor, a City Corporation representative, and a consumer. We used a structured questionnaire to collect quantitative data and conducted key informant interviews. Each key informant interview was audio-recorded and transcribed. Written consent was obtained prior to data collection. To verify the responses of the interviewees, on-site observations were also performed.

### 2.3 Stakeholder workshop

Upon completing the key informant interviews, we held a day-long workshop with participants from various stakeholders involved in TWLAMs. We invited experts from both government and private sectors specializing in human health, animal health, food safety, zoonotic diseases, agriculture, consumer rights, and market regulation. A total of 55 participants attended the workshop, including 16 experts, 10 poultry vendors, 10 market committee members, 10 consumers, and 9 public representatives or respective officials. During the workshop, we shared the results from the key informant interviews for further discussion. Key topics such as market regulations, infrastructure, biosecurity measures, food inspection, and the impact of COVID-19 were discussed in focus group sessions. Each group consisted of 14 members who shared knowledge, experiences, and recommendations.

### 2.4 Data analysis

We categorized the collected data into different sections including market management, infrastructure, hygiene, the role of local government authorities, the risk of zoonotic disease transmission, and the impact of COVID-19. Frequency and percentage were calculated for categorical variables. Data from key informant interviews and the focus group discussion were coded, summarized in a spreadsheet, and highlighted with noteworthy quotes from respondents.

### 2.5 Ethical approval

This protocol was reviewed and approved by the Institutional Review Board of icddr,b (PR-23090). We invited study participants aged over 18 years of age. All the respondents asked to provide informed, written consent to participate in this study.

## 3 Results

We conducted interviews with a total of 40 key informants across 10 selected TWLAMs, all of whom were male. On average, each TWLAM had 190 shops. The average distribution of poultry, fish, meat, vegetable, and grocery shops per market were as follows: 23 poultry shops, 11 fish shops, 55 meat shops, 38 vegetable shops, and 42 grocery shops (Supplementary Table 1).

### 3.1 Ownership and operational guidelines

Of the 10 surveyed TWLAMs, five markets (50 %) were under the governance of the City Corporation, while three markets (30 %) were managed by private owner/companies. The majority of TWLAMs (70 %, *n* = 7) had a functioning management committee. Typically, each market committee comprised 10–15 designated individuals, with an additional 20–30 active members involved. According to respondents, 60 % (*n* = 6) of TWLAMs had written operational guidelines, whereas 40 % (*n* = 4) TWLAMs were unaware of any government directives. Among the 40 key informants, 20 (50 %) preferred the selection process of market committee members over an election process, citing local market leaders' influence in the selection (Table 1). *A respondent from a* TWLAM *said that*

“*Our committee is elected through a selection process. All the members of the market enthusiastically discussed with senior, junior and shopkeepers and selected the committee through selection.*”

(Key informant, market committee member from a market under Gazipur City Corporation)

**Table 1:** Ownership and management systems of wet market, October–December 2023 (*N* = 10).

Characteristics	Number of markets (%)
Market ownership	
City corporation	5 (50)
Private Ownership	3 (30)
Both	2 (20)

Existence of committee	
Yes	8 (80)
No	2 (20)

Functionality of the committee	
Active	7 (70)
Inactive	1 (10)

Committee formation process	
Selection	6 (60)
Election	2 (20)

Presence of guideline to operate market	
Yes	8 (80)
No	2 (20)

Awareness about government instruction on market operation	
Aware	6 (60)
Unaware	4 (40)

The main duties of the market committee included ensuring safety and security, addressing regulatory matters, managing utilities such as power and water, overseeing cleanliness in shared spaces, and monitoring the pricing and quality of goods. Government notices and updates were typically conveyed verbally during committee meetings.

In our observations, live birds, meat, fish, vegetables, and groceries were positioned in clustered arrangements without following any specific pattern. Similarly, participants in the study voiced concerns about notable irregularities in stall positioning, inadequate drainage, and insufficient water supply facilities. Out of the 40 respondents, 14 (35 %) expressed dissatisfaction with the inadequate water supply system. Furthermore, the majority of markets (80 %) did not have direct water supply lines for their shops. *A respondent from a* TWLAM *said that*

“*We have no direct supply line in our market. We have to buy water from other sources at a cost of 50 Bangladesh taka* (*0.42 US Dollars*) *per stall per day.*”

(Key informant, vendor from a market under Dhaka South City Corporation)

### 3.2 Biosecurity practices of poultry shops

The infrastructure of poultry shops varied from one market to another. According to study participants, infrastructure was well-organized in six markets but disorganized in two markets. Forty percent (*n* = 4) of markets experienced inadequate water supply and 50 % (*n* = 5) markets had no functional drainage systems. Cleaning was regularly conducted at the shop level by shop workers, though the entire market cleaning was infrequent (Table 2). City Corporation staff were not directly engaged in market cleaning. *A respondent from a* TWLAM *said that*

“*Our market committee has assigned two workers to clean the market. They arrive at noon and again at night after the market closes. All the shop owners collectively pay the workers' wages on a monthly basis.*”

(Key informant, vendor from a market under Dhaka North City Corporation)

**Table 2:** Infrastructure, cleaning, disinfection and waste disposal practices of wet markets, October–December 2023 (N = 10).

Characteristics	Number of markets (%)
Position of different categories of shops within market	
Organized	6 (60)
Fairly Organized	2 (20)
Disorganized	2 (20)

Drainage system	
Well-functioning	5 (50)
Partial-functioning	2 (20)
Not functioning	3 (30)

Water supply	
Adequate	6 (60)
Partial	2 (20)
No supply	2 (20)

Water source	
Pipe water supplied by Water and Sewerage Authority (WASA)	7 (70)
Stored water in bucket collected from centrally placed water pipe	2 (20)
Collected from outside source	1 (10)

Frequency of entire market cleaning	
Daily	2 (20)
Sometimes but not daily	4 (40)
Never	4 (40)

Cleaning and disinfection	
Use only water	4 (40)
Use water with disinfectants (bleaching powder, detergent, soap)	2 (20)

Person involved in waste disposal	
Recruited by market committee	3 (30)
Recruited by City Corporation	6 (60)
Recruited by individual shop owner	1 (10)

Frequency of waste disposal	
Once a day	9 (90)
Twice a day	1 (10)

City Corporations employ veterinary inspectors, slaughterhouse inspectors, and sanitary inspectors responsible for monitoring food quality and market cleanliness. According to respondents, waste disposal is a primary activity managed by City Corporations. A majority (55 %, *n* = 22) of respondents reported not noticing any food inspectors at the market (Supplementary Table 2). Occasionally, mobile courts, overseen by judicial officials under higher judiciary supervision, make unannounced visits to investigate the food quality and pricing of goods, but these were rarely seen. Cleaners employed by the City Corporation collect waste once daily using trash vans, transporting it to the nearest garbage disposal containers. In markets not regulated by the City Corporation, privately recruited workers handle waste management. A significant number of respondents (37 %) noted that workers in markets typically do not wash their hands (Table 3). Key challenges in wet markets were reported to include cleaning, poor infrastructure, inadequate drainage, and limited space. Most respondents emphasized the need for infrastructure development of TWLAM, enhanced cleaning practices, and improved waste disposal methods (Table 4).

### 3.3 Knowledge about zoonotic diseases transmission and impact of COVID-19

Among the 40 respondents, only 60 % (*n* = 24) believed that live animals can transmit diseases to humans within wet markets. Similarly, only 55 % (*n* = 22) of respondents indicated that humans could become infected through contaminated water within wet markets. According to self-reports, COVID-19 had various impacts on TWLAM including food supply, food prices, market hygiene, personal hygiene, and livelihoods. The most common negative impacts of COVID-19 in wet markets were increased living expenses (67 %) and job losses (65 %). On the positive side, COVID-19 led to improvements in market hygiene (100 %), personal hygiene (100 %), and adherence to social distancing (100 %). No permanent closures or bans on animal trading were reported in the wet markets during COVID-19 pandemic. While social distancing and mask-wearing were practiced to some extent, their implementation was not always consistent or effective (Table 5).

**Table 3:** Cleaning, disinfection and waste disposal practices of wet markets.

Characteristics	Number of markets (%)
Frequency of entire market cleaning	
Daily	2 (20)
Sometimes but not daily	4 (40)
Never	4 (40)

Cleaning and disinfection	
Use only water	4 (40)
Use water with disinfectants (bleaching powder, detergent, soap *etc.*)	2 (20)

Person involved in waste disposal	
Recruited by market committee	3 (30)
Recruited by City Corporation	6 (60)
Recruited by individual shop owner	1 (10)

Frequency of waste disposal	
Once a day	9 (90)
Twice a day	1 (10)

**Table 4:** Personal hygiene and knowledge on zoonotic disease transmission (*N* = 40).

Characteristics	Number of respondents (%)
Practice of hand wash among wet market workers	
Frequently	8 (20)
Sometimes	13 (32)
Occasionally	4 (10)
Lack	15 (37)

Participated in biosafety and hygiene training	
Yes	16 (40)
No	24 (60)

Perceived that live animals transmit diseases to humans within wet markets	
Yes	24 (60)
No	16 (40)

Perceived that humans can be infected with contaminated water within wet markets	
Yes	23 (57)
No	17 (43)

**Table 5:** Perception towards wet market and live animal trading among 40 key informants in Bangladesh.

Characteristics	Number of respondents (%)
Good things about live animals and wet market	
Fresh foods	28 (70)
Relatively affordable	20 (50)
Available goods	13 (32)
Consumer's comfort	6 (15)
Car parking facility	4 (10)
Business independency	2 (5)
Clean market	4 (10)

Major challenges in the wet markets	
Lack of cleanliness	8 (20)
Poor road condition	4 (10)
Waterlogging	3 (7)
Lack of space for car parking	3 (7)
Load unload issue	3 (7)
Disorganized	2 (5)
Limited space	2 (5)
Litigation issue	2 (5)
Construction issue	2 (5)

Recommendation to improve wet markets	
Infrastructure development	20 (50)
Proper cleaning and waste disposal	8 (20)
Road construction	7 (17)
Space allocation	3 (7)
Laws and regulation implementation	2 (5)
Slaughterhouse, increase manpower	1 (2)

### 3.4 Summary of TWLAM stakeholder's workshop findings

In the stakeholder's workshops, participants identified numerous challenges and gaps affecting TWLAMs. These included difficulties in implementing laws and guidelines, the absence of uniform market standards, specific regulations for poultry trading, and low awareness of existing regulations. Political interference, issues with food certification and financial management, ineffective market committee operations, as well as insufficient space for shops and narrow walkways were also highlighted. Other concerns encompassed overcrowding, poor drainage, inadequate water supply, traffic congestion, lack of a central slaughter area, and sanitation problems. The proliferation of mobile poultry vendors, inadequate monitoring of market operations, and the absence of designated areas for sick animals were also noted. Participants raised issues such as the lack of incentives for good practices, insufficient punishment for mismanagement, limited handwashing facilities, absence of uniforms for butchers or poultry workers, and inadequate hygiene knowledge. They also highlighted challenges related to environmental contamination, limited disinfectant supply, irregular cleaning by City Corporation staff, and manpower shortages for cleaning and waste disposal. Additionally, concerns were expressed about the inadequate number of food inspectors, poor consumer awareness regarding food quality, and insufficient coordination among market stakeholders. The absence of mechanisms for consumer complaints or feedback, as well as reluctance to adhere to respiratory etiquette, were also cited as issues. Regarding the shift from live animal trading to processed animal products, participants acknowledged cultural norms and practices as significant barriers. However, they believed that with adherence to standard protocols and the establishment of consumer trust, such a transition could be successful. There was consensus among participants that transitioning to processed animal products could reduce the transmission of zoonotic diseases from animals to humans. They unanimously requested government support in budgeting, certification, training, and sales promotion of food products (Table 6).

**Table 6:** Impact of COVID-19 on food security, safety and livelihood (N = 40).

Characteristics	Number of respondents (%)
Impact of COVID-19 on food security	
Inadequate supply of food	12 (30)
Adequate supply of food	28 (70)
Increase food price	8 (20)
No impact on price	32 (80)

Impact of COVID-19 on market and personal hygiene	
Enhanced market hygiene	40 (100)
Enhanced monitoring of food quality	17 (42)
Maintained social distance	40 (100)
Enhanced personal hygiene	40 (100)

Impact of COVID-19 on livelihood	
Employment lost	26 (65)
Increased living expense	27 (67)

## 4 Discussion

Creating a standardized TWLAM system requires integrating multiple essential elements, including effective policy implementation, regulations, guidelines, infrastructure improvements, enhanced biosecurity practices, financial support, and the promotion of food safety. This study focused on assessing the enforcement of regulations, the availability of operational guidelines, and the roles played by market committees in traditional wet and live animal markets. These markets were overseen either by local governments or private companies. There was a lack of uniform guidelines or standard operating procedures (SOPs) for wet markets, with many stakeholders unaware of existing policies. Establishing a consistent practice in wet markets to ensure a safe food supply could be facilitated by government regulatory bodies developing standardized operational guidelines for wet and live animal markets.

In Bangladesh, the “Hats and Bazars (Establishment and Acquisition) Act, 2023” primarily governs the administrative procedures for formalizing wet, village, animal, and food markets [6]. The main responsibilities of this committee involve collecting tolls regularly, maintaining law and order, preventing illegal activities, and ensuring sanitary facilities. The central government collects revenue from value-added tax, income tax, and land tax from individual shops, the committee, or the market leaseholder [5]. There is no specific legislation governing the daily operations of wet markets that specifically addresses issues such as biosecurity, personal hygiene, and the risk of zoonotic exposure. Similar to Bangladesh, other Asian countries have regulations governing the operation of wet markets. In India, the Food Safety and Standards Act (FSSA), 2006, introduced a regulatory framework called “The Food Safety and Standards (Licensing and Registration of Food Businesses) Regulations, 2011,” which aims to license and register food businesses, including those involved in meat and meat-based products [11]. In the Philippines, wet markets are regulated by local government ordinances under Republic Act No. 7160. The main activities include the collection of taxes and rents from stalls and spaces within the markets [12]. Regulations for wet markets in many countries primarily focus on tax collection and stall management, with less emphasis on promoting food safety, safer food processing, hygiene, and biosecurity practices. The recent emergence of COVID-19 has highlighted the need to update wet market regulations to enhance biosecurity measures and ensure continuous monitoring, however, according to our results, there seems to have been no impact of COVID-19 in this regard yet.

The wet markets evaluated in this study exhibited significant variation in infrastructure, number of shops, types of shops, cleaning practices, disinfection, water supply, and waste disposal. Specific operational guidelines for biosecurity practices and infrastructure were notably absent. Many of the markets faced challenges such as inadequate water supply and deficient drainage systems. Previous research has often concentrated on biosecurity practices within poultry shops, overlooking comprehensive assessments of entire markets encompassing diverse types of food shops [[13], [14], [15]]. The floor surfaces in many live bird markets and shops have been found to be uneven, and a significant number of shops did not conduct regular cleaning and disinfection [13,14]. Wet markets with rough dirt and mud floors reflect poor infrastructure, which hinders effective cleaning and disinfection [14]. This study and other previous studies identified several key areas for the improvement. A periodical risk assessment of every individual wet market may provide valuable insights to the market authorities for necessary actions.

Though wet markets are important sources for fresh foods, live animal trading and slaughtering can facilitate transmission of zoonotic pathogens from animal to humans. Our study also identified many respondents who perceived that live animals transmit pathogens to humans within wet markets. However, the majority of workshop participants found it challenging to transition from trading live animals to animal products due to cultural habituation. People living in Bangladesh prefer to buy live animals and have them slaughtered in front of them. The majority of people have low trust in ready-to-sell slaughtered or frozen poultry, believing that businessmen might slaughter and sell sick or dead poultry. Like Bangladesh, selling live poultry and animals are common in many Asian countries including China, Vietnam, Indonesia and Cambodia [[16], [17], [18], [19]]. The wet market regulatory authorities in Bangladesh should enhance monitoring of the implementation of improved biosecurity measures and regular food inspections until live poultry trading is phased out. On a positive note, the findings of our study show that most key informants did perceive the challenges of poor infrastructure and hygiene, and may be willing to do improvements, given the right resources and incentives.

Live and wet markets were identified as important sources of several emerging and re-emerging pathogens, including various avian influenza virus subtypes and foodborne pathogens [1]. Most of the earliest COVID-19 cases were linked to a market in China, suggesting that SARS-CoV-2 likely spread from wild animals kept at the market to humans [20]. The recent COVID-19 pandemic had several impacts including public health, live animal market and livelihood [[21], [22], [23]]. Many wet markets in China were either completely banned or partially closed for a period to help reduce the spread of SARS-CoV-2 and H5N1 virus [24,25]. Although COVID-19 raised concerns about the role of biosecurity in wet markets to prevent the emergence of pandemic-potential zoonotic pathogens, no studies have explored the positive impact of COVID-19 in facilitating biosecurity improvements, reform efforts and policy in these markets [26,27]. Like other countries, COVID-19 caused serious threat to human health in Bangladesh [28]. Our research revealed the impact of COVID-19 on food supply, hygiene practices, and livelihoods in the traditional wet markets of Bangladesh. During COVID-19, living expenses of market workers were increased and led to job losses. Previous studies from Bangladesh indicated that during lockdowns, the prices of essential food items such as rice and potatoes increased by up to 23 % and 43 %, respectively, while the average price of broiler chicken meat decreased by up to 13 % [29,30]. Regarding chicken prices, India experienced a notable increase of up to 29.9 % due to reduced availability during COVID-19 [31]. Wild animal trading in Wuhan, China, was permanently prohibited [32], but in Bangladesh there were no closures. According to the International Monetary Fund (IMF), global consumer food prices increased by 2 % to 9 %, depending on the type of food product, due to COVID-19 [33]. Finding of the studies suggest wet markets and their associated activities can be affected because of pandemics and poor market regulations. However, phasing out live animal sales at traditional wet markets may not be feasible in Bangladesh due to socio-economic impacts. Developing operational guidelines and regulations for wet markets is crucial to ensure proper daily activities, maintain personal hygiene, implement biosecurity measures, and improve infrastructure.

## 5 Conclusion

This study identified several gaps, limitations, and challenges in implementing standard operational procedures to ensure the supply of safe foods to consumers, as well as to reduce risks of other zoonotic transmission. The operating systems of the studied markets were unsystematic, and many lacked operational guidelines. Stall positions by category were poorly organized, and biosecurity practices were below standard. The government may need to emphasize developing a unique operational guideline through financial incentives, certification, training, and regular monitoring.

The following are the supplementary data related to this article.

Supplementary Table 1 Market wise shop number in 10 selected wet markets located in Dhaka and Gazipur City Corporation areas. Supplementary Table 1

Supplementary Table 2 Role of city corporation and other government departments (N = 40). Supplementary Table 2

## CRediT authorship contribution statement

**Sukanta Chowdhury:** Writing – review & editing, Writing – original draft, Validation, Project administration, Methodology, Investigation, Funding acquisition, Conceptualization. **Tushar Kumar Das:** Writing – review & editing, Formal analysis. **Nurun Nahar Chisty:** Writing – review & editing, Formal analysis. **Sajal Kanti Biswas:** Writing – review & editing, Formal analysis. **Mohammed Ziaur Rahman:** Writing – review & editing, Formal analysis. **Jiaxin Ling:** Writing – review & editing, Validation, Project administration, Methodology, Investigation, Funding acquisition, Conceptualization. **Mahmoud M. Naguib:** Writing – review & editing, Validation, Project administration, Methodology, Investigation, Funding acquisition, Conceptualization. **Johanna F. Lindahl:** Writing – review & editing, Validation, Project administration, Methodology, Investigation, Funding acquisition, Conceptualization.

## Funding

This study was funded by the 10.13039/501100004359Swedish Research Council for Sustainable Development Formas 2021-00833.

## Declaration of competing interest

The authors declare that they have no known competing financial interests or personal relationships that could have appeared to influence the work reported in this paper.

## Data Availability

The data from this study are available from the corresponding author upon reasonable request.
