# Supplementary material for: Regulations, biosecurity measures, and impact of COVID-19: A comprehensive mixed method study in traditional wet and live animal markets in Bangladesh
Source: One Health. 2025 Mar 17;20:101014. doi: 10.1016/j.onehlt.2025.101014 (PMC11979392; doi:10.1016/j.onehlt.2025.101014)
Supplement: Supplementary Table 1 — Market wise shop number in 10 selected wet markets located in Dhaka and Gazipur City Corporation areas. [file mmc1.docx]

**Supplementary Table 1:** Market wise shop number in 10 selected wet markets located in Dhaka and Gazipur City Corporation areas

| **Study areas** | **Market Name** | **Poultry Shop** | **Meat Shop** | **Fish shop** | **Vegetable Shop** | **Stall selling food that is ready-to-eat** | **Groceries** | **Others** | **Total shops** |
| --- | --- | --- | --- | --- | --- | --- | --- | --- | --- |
| Dhaka South City Corporation | Market-1 | 22 | 3 | 34 | 39 | 4 | 35 | 8 | 145 |
|  | Market-2 | 24 | 7 | 33 | 7 | 6 | 20 | 10 | 107 |
|  | Market-3 | 14 | 7 | 12 | 19 | 7 | 30 | 23 | 112 |
| Dhaka North City Corporation | Market-1 | 14 | 8 | 31 | 25 | 15 | 13 | 6 | 112 |
|  | Market-2 | 18 | 20 | 63 | 55 | 9 | 45 | 34 | 244 |
|  | Market-3 | 30 | 22 | 71 | 39 | 5 | 82 | 12 | 261 |
|  | Market-4 | 36 | 7 | 70 | 42 | 2 | 80 | 24 | 261 |
| Gazipur City Corporation | Market-1 | 23 | 15 | 105 | 64 | 6 | 58 | 12 | 283 |
|  | Market-2 | 32 | 19 | 64 | 44 | 3 | 16 | 7 | 185 |
|  | Market-3 | 14 | 7 | 70 | 50 | 9 | 40 | 2 | 192 |
| **Total (%)** | | **227 (12)** | **115 (6)** | **553 (29)** | **384 (20)** | **66 (4)** | **419 (22)** | **138 (7)** | **1902** |
| **Average number of shops** | | **23** | **12** | **55** | **38** | **7** | **42** | **14** | **190** |
