# Supplementary material for: Regulations, biosecurity measures, and impact of COVID-19: A comprehensive mixed method study in traditional wet and live animal markets in Bangladesh
Source: One Health. 2025 Mar 17;20:101014. doi: 10.1016/j.onehlt.2025.101014 (PMC11979392; doi:10.1016/j.onehlt.2025.101014)
Supplement: Supplementary Table 2 — Role of city corporation and other government departments (N = 40). [file mmc2.docx]

**Supplementary Table 2.** Role of City Corporation and other Government Departments (N=40)

| **Characteristics** | **Individual level response (%)** |
| --- | --- |
| **Regular activities of City Corporation** **and other Government Departments** | |
| Waste disposal  Traffic control  Food inspection  No regular activities | 22 (55)  2 (5)  3 (7)  13 (32) |
| **Regular communication by City Corporation** **and other Government departments** | |
| Yes  No | 12 (30)  28 (70) |
| **Noticed sanitary/food inspectors within last three months** | |
| Yes  No | 18 (45)  22 (55) |
| **Frequency of Inspector visits**  Daily  Weekly visit  Monthly visit  Occasionally | 3 (8)  2 (5)  4 (10)  9 (23) |
| **Common activities of inspectors**  Inspect live animal before and after slaughtering  Inspect food quality  Inspect food price  Advise shop owners | 4 (10)  7 (17)  9 (22)  5 (12) |
